# The Influence of Manufacturing Parameters on the Mechanical Behaviour of PLA and ABS Pieces Manufactured by FDM: A Comparative Analysis

**DOI:** 10.3390/ma11081333

**Published:** 2018-08-01

**Authors:** Adrián Rodríguez-Panes, Juan Claver, Ana María Camacho

**Affiliations:** Department of Manufacturing Engineering, Universidad Nacional de Educación a Distancia (UNED), Madrid 28040, Spain; adrian.rodriguez@invi.uned.es (A.R.-P.); jclaver@ind.uned.es (J.C.)

**Keywords:** additive manufacturing, FDM, polylactide (PLA), acrylonitrile butadiene styrene (ABS), tensile behaviour, layer height, infill density, layer orientation

## Abstract

This paper presents a comparative study of the tensile mechanical behaviour of pieces produced using the Fused Deposition Modelling (FDM) additive manufacturing technique with respect to the two types of thermoplastic material most widely used in this technique: polylactide (PLA) and acrylonitrile butadiene styrene (ABS). The aim of this study is to compare the effect of layer height, infill density, and layer orientation on the mechanical performance of PLA and ABS test specimens. The variables under study here are tensile yield stress, tensile strength, nominal strain at break, and modulus of elasticity. The results obtained with ABS show a lower variability than those obtained with PLA. In general, the infill percentage is the manufacturing parameter of greatest influence on the results, although the effect is more noticeable in PLA than in ABS. The test specimens manufactured using PLA perform more rigidly and they are found to have greater tensile strength than ABS. The bond between layers in PLA turns out to be extremely strong and is, therefore, highly suitable for use in additive technologies. The methodology proposed is a reference of interest in studies involving the determination of mechanical properties of polymer materials manufactured using these technologies.

## 1. Introduction

Additive manufacturing (AM) encompasses numerous technologies that allow for the construction of three-dimensional parts by superimposing layers of material. These techniques have undergone great development in recent years. The most widespread group of these is Material Extrusion manufacturing. These processes have the great advantage of low equipment and material costs, ease of use in laboratories and domestic environments, and the versatility to manufacture all kinds of shapes with a wide range of materials, mainly plastics, in a short time. Additive manufacturing enables the obtaining of extremely complex geometries in a single process, also with regard to interior cavities, thus providing great freedom during the design stage [1]. On the other hand, the development of new technologies and commercially available equipment for additive manufacturing using different materials, such as metals [2] and composites, brings the pieces obtained ever closer to the optimum values, not only with respect to performance in service, but also with respect to dimensional and geometrical precision [3]. Furthermore, this type of processes also provides advantages and new challenges [4] from the point of view of sustainability [5,6] and design optimisation [7,8]. Additive manufacturing is constantly developing and talk is already afoot regarding advanced concepts such as 4D printing, reflected by Momeni et al. in their 2017 review [9]. Its use is also being extended to applications in medicine [10] and even within the area of university teaching, as shown in studies such as that carried out by García-Domínguez et al. [11].

**Figure 1:**
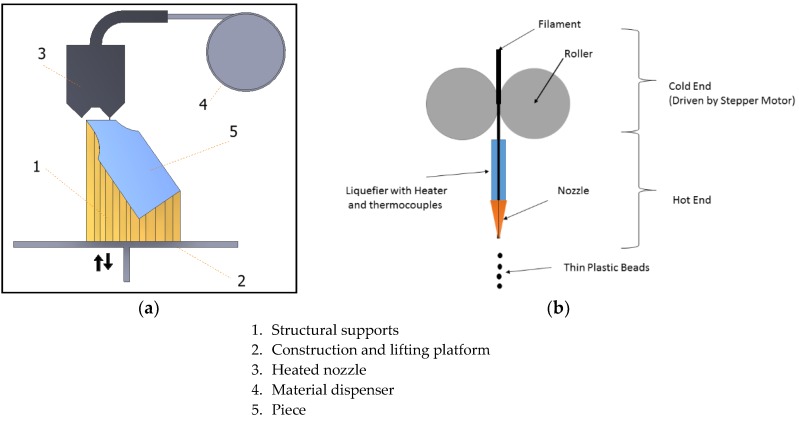
(**a**) A sketch of the material extrusion process; (**b**) An extruder sketch in an FDM/FFF (Fused Deposition Modelling/ Fused Filament Fabrication) process.

The origin of the concept goes back to the 1980s with “Stereolithography” [12] and a whole host of new techniques have been developed since then, as explained by Gibson et al. in their book released in 2015 [13]. One of the categories or typologies acknowledged both in works undertaken by diverse authors [12] and in the recent ISO 17296-2:2015 standard on Additive Manufacturing [14] is Material Extrusion (Figure 1). This category encompasses processes in which the material, usually thermoplastic, is selectively applied using a nozzle to form each layer. The most important commercial process is known as Fused Deposition Modelling (FDM), which was patented by the founder of Stratasys over 20 years ago. Another similar process, but one that is not subject to this patent, is Fused Filament Fabrication (FFF).

When compared with conventional manufacturing processes, one of the main problems to be tackled is that the traditional test procedures cannot always be applied to additive manufacturing processes. As opposed to the traditional processing of polymers, layer-based production generates parts with anisotropic properties and residual stresses. For this reason, standardised methods are required that enable the linking of the properties of the material, the manufacturing parameters and the design of the piece, which represents a major challenge. Given the emerging nature of some of the technologies and their rapid development, the literature available in this area is not very extensive. However, a number of works have recently been published devoted to the mechanical characterisation of parts produced using FDM, such as that of Goh et al. [15], on reinforced thermoplastics; that of Domingo et al. [16], on using polycarbonate as the material; that of Mohamed et al. [17], where the effect of the process conditions on the temperature-dependent dynamic mechanical properties of processed PC-ABS (polycarbonate-acrylonitrile butadiene styrene) parts by FDM was analysed; that of Tymrak et al. [18], focusing on ABS and PLA (polylactide); and that of Chacón et al. [19], which concentrates on studying the influence of the manufacturing parameters on the mechanical properties of PLA parts, all of which show the interest of the scientific community to increase the knowledge about this subject.

Standard ISO17296-3 [20] covers the main surface, geometrical and mechanically requirements depending on the material (metal, plastic, or ceramic) and on the criticality ratings of the parts (highly engineered parts, functional parts that are not safety critical and prototype pieces). It also indicates the standards to be used to determine the principal quality characteristics and the corresponding test standards, not only for the starting material, but also for the pieces produced. In this respect, the most appropriate test standard is indicated for determining the mechanical requirements of the plastic pieces produced using additive manufacturing, with ISO 527-2 [21] being specified for the tensile strength. It sets forth the tensile test conditions for rigid and semi-rigid extruded plastics, which is the closest example to the pieces that form the objective of this study. For its part, standard ISO/ASTM 52921-13 [22] specifies the nomenclature and terms associated with coordinate systems and testing methodologies for additive manufacturing. Additionally, on the other hand, standard ASTM D638-14 [23] establishes a test method for determining the tensile properties of reinforced and unreinforced plastics. The problem with all of these guidelines is that they refer to test procedures that still do not include specific considerations for AM. This lack of specific regulation with respect to additive manufacturing represents a serious problem as it makes it difficult to establish valid comparisons between machines, materials, and models that make it possible to predict the properties of the final pieces and establish design guidelines.

In this respect, Forster [24] collates the existing procedures for the testing of polymers and analyses their viability for additive manufacturing processes. The aforementioned standards ISO 527 [21] and ASTM D638-14 [23] can be consulted for the profiling of tensile properties. These standards are recognised as being valid for additive manufacturing processes, although amendments might be required with respect to the post-processing of the test specimens in order to meet the demands of the standard (surface finish or dimensional requirements) or their applicability might be limited as they do not meet the material isotropy requirements [25]. Although the methods for isotropic materials can be applied, this would lead to the results obtained being more uncertain and it will not be possible to equate the properties obtained for the material with those of the specific piece.

As claimed by Tymrak et al. [18], in order for FDM printed parts to be useful for engineering applications, the mechanical properties of parts produced by this technique must be known. Forster identifies a number of geometrical variables of the deposition of material with an influence on the mechanical properties of the piece, such as raster angle, the height and width of the layer, the space between extruded filaments, the combination of variables (space between extruded filaments, layer width and height or deposition velocity) that might increase the overlap between filaments, or the orientation of the piece during manufacture (which can affect the transfer of load between filaments and layer interfaces).

There are previous studies that have analysed the influence of some of these parameters for certain materials. Many of these focus on the influence of raster angle [26], a parameter of the process that significantly affects the anisotropy and strength of the pieces. According to the study undertaken by Rodríguez et al. [27], this parameter causes variations in Young’s modulus between 11% and 37%. The majority of these works show that the parts are stronger when the lines are oriented in the load direction for tensile tests [28,29,30], mixed angles for flexion [28,31], and orthogonal to the yield load [32]. The higher the raster angle, the lower the tensile properties of the material [27,28], with these reaching a minimum of around 50° [33].

The mechanical strength of a piece produced using AM is always lower than that of the original material or that of injection moulded pieces [34]. However, 80% of an injection moulded piece’s strength can be achieved by orienting the lines in the direction of the load [35]. Fernández-Vicente et al. [36] carried out a study that analyses the influence of the pattern and infill percentage, with the conclusion being that the influence of the different print patterns produces a variation of at least 5% in the maximum tensile strength, meaning that the performance is similar. On the other hand, the density of the infill is a decisive factor in tensile strength; the combination of a rectilinear pattern and a 100% infill provides the greatest tensile strength with a value of 36.4 MPa and a difference of less than 1% with respect to the filament (ABS).

Regarding the influence of the space between filaments, the presence of cavities and sharp corners increases the stress within the piece, which may cause failure [21]. In general, minimising the space between the filaments increases the contact area between them and lead to a stronger fusion interface. The material extrusion processes depend on the temperature gradients between contiguous filaments as these enable the thermoplastic polymers to form a solid fusion interface. Different studies set out to establish manufacturing guidelines related to the deposition speed and temperature and the temperature of the chamber which enable the production of stronger joints and, consequently, improved mechanical properties [32,37]. These type of studies enables the manufacturing parameters, the design of the parts and the final properties to be linked. Narrowing the width of the lines extruded reduces the residual stress in the filament and can increase the diffusion length. However, this would require more passes to create the piece, which increases the residual stress caused by the contraction of the polymer during cooling and, furthermore, the successive changes of nozzle speed have negative consequences in the diffusion [28].

With respect to manufacturing direction, Riddick et al. [29] combined orientations *xz*, *yz*, and *xy* with various raster angles (0°, 0°/90°, and 90°) and found that orientation *xz* had the highest modulus of elasticity, (*E* = 2.67 GPa) and the greatest tensile strength (15.26 MPa). Other authors have verified that the strength is increased by maximising the alignment of the layers in the load direction [31].

On the other hand, the manufacture of pieces using FDM/FFF processes is subject to numerous variables dependent upon thermophysical and/or chemical phenomena that are going to result in pieces with different characteristics depending on the method used along with the parameters of the process. One of these important phenomena is the inter-molecular diffusion between layers and/or dissimilar materials that influences the interfacial bonding strength, as explained in the work by Yin et al. [38] where inter-molecular diffusion theory based on heat transfer is developed and the influence of processing parameters on bonding strength has been investigated. A significant improvement in layer adhesion and a more isotropic part was obtained by Levenhagen and Dadmun [39] by developing a process in which bimodal blends of the same polymer with different molecular weight were used. Another interesting approach is the one presented by Ravi et al. [40], consisting of the development of a pre-deposition heating method to heat the region of an existing layer before the new layer is deposited; thus, the temperature at the inter-layer interface increases, improving the interpenetrating diffusion, leading to a better bond strength. Particularly for polymer-fibre composites but not limited to, other aspects including void formation, blockage due to filler inclusion and/or poor adhesion of fibres and matrix must be addressed in the close future, as stated by Parandoush and Lin in their work from 2017 [41]. The complexity of these processes due to the high number of parameters involved and their interdependencies requires multidisciplinary research [42]. Something that further complicates the analysis of the mechanical performance of these pieces is that they are normally fragile and fracture easily due to interlaminar failure caused by manufacturing defects that are difficult to control [15]. The nature of these phenomena is very complex, as they are also affected by the influence of related environmental and process conditions; for example, if the process is performed within a controlled atmosphere, the existence (or the lack thereof) of a heated bed and/or the heat transmission process affects the thermal gradients of the workpiece, particularly between layers.

Our study is focused on analysing more global trends for a wide range of parameters, including the most used values in the practice, independently of the equipment. The main interest of these parameters is that they can be accurately defined as they are strictly “manufacturing parameters” and they can be reproduced in all the FDM equipment independently of the technology used so that they can be reproducible for third parts in different application fields.

This paper sets out to establish a relationship between the manufacturing parameters and the mechanical properties of the piece by using the test procedures set forth in the existing standards on plastics for the polymer materials most used in FDM techniques: acrylonitrile styrene butadiene (ABS) and polylactide (PLA). These, together with nylon, polyethylene (PET, PETG), polycarbonate (PC), and thermoplastic elastomers (TPE) are commonly used for prototyping design and the creation of low-performance parts. This comparative study will enable us to know more about the mechanical performance of ABS and PLA pieces manufactured using FDM in terms of the main manufacturing parameters: the influence of layer height, the percentage of infill and the orientation of the object during manufacture.

## 2. Materials and Methods

### 2.1. Materials and Equipment

This paper analyses the mechanical performance of pieces manufactured using PLA and ABS, the most commonly used materials in material extrusion 3D printing technologies. PLA is a biodegradable polymer derived from lactic acid. The main advantage of this material is how easy it is to use in 3D printing and the good results it delivers. It requires a lower extrusion temperature than ABS, it does not suffer significant distortions during printing and it adheres well to the platform, which means it does not require a heated base. Neither does it give off a bad smell or toxic vapours during printing. It must not be used for parts that have to withstand high temperatures because PLA tends to warp at over 60 °C. ABS is a thermoplastic that is extremely resistant to impact, abrasion, and chemical elements. In 3D printing, it is the most used material after PLA. Its good mechanical properties, resistance to temperature, low price, moderate flexibility, long service life and range of melting temperatures make this material an excellent option for manufacturing all manner of parts using FDM technologies, above all, parts that have to withstand cyclical loads and temperature changes. However, it is not suitable for all applications as it presents problems of contraction and warping during printing, tends to peel away from the platform, and tends to give off toxic gases. Table 1 shows the data provided by the manufacturer for the filaments of the two materials used. As can be seen, PLA is more rigid and has a greater tensile strength while ABS is more ductile. However, the impact strength of ABS is far greater (320 J/m against 220 J/m (Notched Izod Impact)), one of the main properties that differentiates it from other plastics.

**Figure 2:**
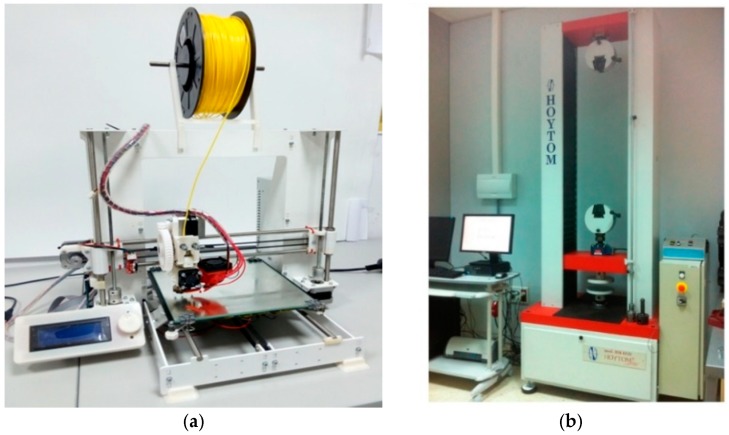
The equipment: (**a**) a Prusa i3 printer; (**b**) a HOYTOM HM-D 100kN Universal testing machine.

The additive manufacturing equipment to be used is the Prusa I3 Aluminium printer (Prague, Czech Republic) (Figure 2a), which employs FFF technology. The Cura software (Ultimaker, Geldermalsen, The Netherlands) is used to export the three-dimensional models of the samples to G-code. The main technical characteristics of the FDM printer are defined in Table 2. The tests are carried out using a HOYTOM HM-D 100 kN model universal testing machine (Leioa, Spain) (Figure 2b).

### 2.2. Mechanical Properties of FDM Filaments

**Figure 3:**
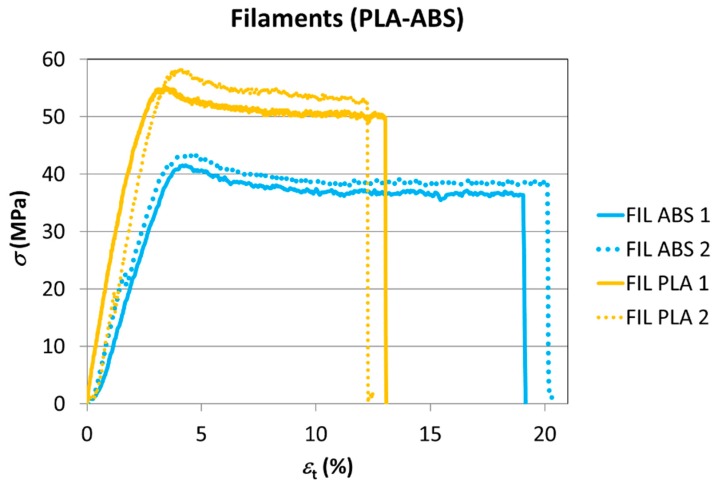
The tensile tests of the filaments.

Mechanical tests were carried out on filaments of the two materials in their raw state to compare the data with those obtained by the manufacturer. Two filaments of every material (ABS 1, ABS 2, PLA 1, and PLA 2) were tested by the authors (Figure 3) and the average values of the main mechanical parameters (tensile strength, nominal strain at break, modulus of elasticity) were compared with those provided by the manufacturer (Table 1) and presented in Table 3; in this case, the manufacturers did not specify how these properties were determined, but standardized specimens of thermoplastic materials for tensile testing are traditionally manufactured by injection moulding or machined. In this work, we want to check how accurate the results from those obtained by the manufacturer by traditional processes by applying tensile tests directly to filaments as they are the starting material for an FDM process. There are no specific standards for this kind of testing due to the novelty of the FDM technique, where pieces are obtained after depositing the filament during a layer by layer operation. The filaments are provided as solid wires of diameter 1.75 mm in coils and the tests were carried out directly by applying tensile forces to filaments of 165 mm in length.

The mechanical properties derived from the tests are shown in Table 3.

As it can be seen, the data obtained for the ABS and the PLA from tensile tests of the filaments are only qualitatively congruent with those provided by the manufacturer; the data that show a higher degree of coincidence are those associated with mechanical tensile strength, particularly results for ABS, that present a very similar value (0.4%); significant differences are found for the other two parameters recorded (nominal strain at break and modulus of elasticity), especially in the case of the material PLA, where differences of 112.5% and 44.6% have been obtained, respectively. This can be due to differences between the procedures followed, as explained before; however, the tensile strengths seem to have a better coincidence among results.

### 2.3. Design of Manufacturing Parameters and Case Studies

**Figure 4:**
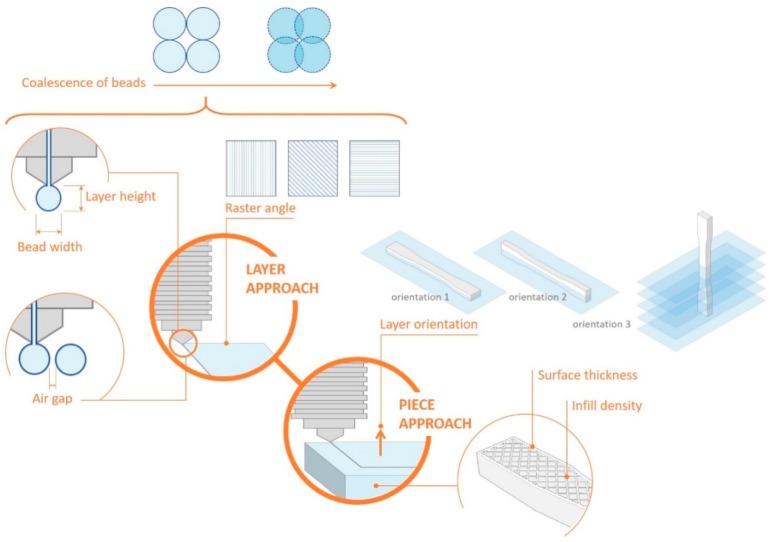
The geometrical parameters with a direct influence on the mechanical properties of pieces manufactured using FDM.

The mechanical characteristics of the pieces produced using AM are very much dependent upon the manufacturing parameters. Figure 4 includes a diagram of the most relevant of those parameters.

This paper will study the influence of layer height, the percentage of infill, and construction orientation of the test specimen on the mechanical tensile properties of the two materials as these are the three most influential parameters. One of the main objectives is to check how much the mechanical performance improves when the percentage of the infill is increased, which is why two values (20% and 50%) have been included in the study. By considering one or the other infill value, the volume ratio of material changes due to the variation of the infill density which applies different sizes of cell patterns. On the other hand, the thickness of the piece’s shell remains constant, with a value of 0.8 mm. Layer height is a parameter that influences manufacturing time; the precision of details and the finish are significantly improved by using finer layers. However, this study sets out to also analyse the influence of this parameter on mechanical strength. To do so, two layer heights, 0.1 mm and 0.2 mm, will be used. Finally, a comparison will be made to discover how much the mechanical strength varies with respect to the orientation of the piece during its construction. This is a fundamental factor due to the anisotropy conferred upon the piece by the layering manufacturing process. Both in the case of using orientation 1 and in that of using orientation 2, the layers are parallel to the stress, but the orientation of the infill raster varies with respect to the tensile force. Furthermore, in this case, it is necessary to use supports, and this causes a certain degree of roughness on the overhanging surface when removing them, but the results are acceptable for test purposes. In the case of orientation 3, layering in a direction perpendicular to the stress should result in a reduction of mechanical strength. A summary of the parameters whose influence on mechanical performance is going to be analysed is presented in Table 4.

Finally, the rest of the parameters are specifically defined for each material in Table 5.

### 2.4. Experimental Procedure and Geometrical Dimensions of Test Specimens

**Figure 5:**
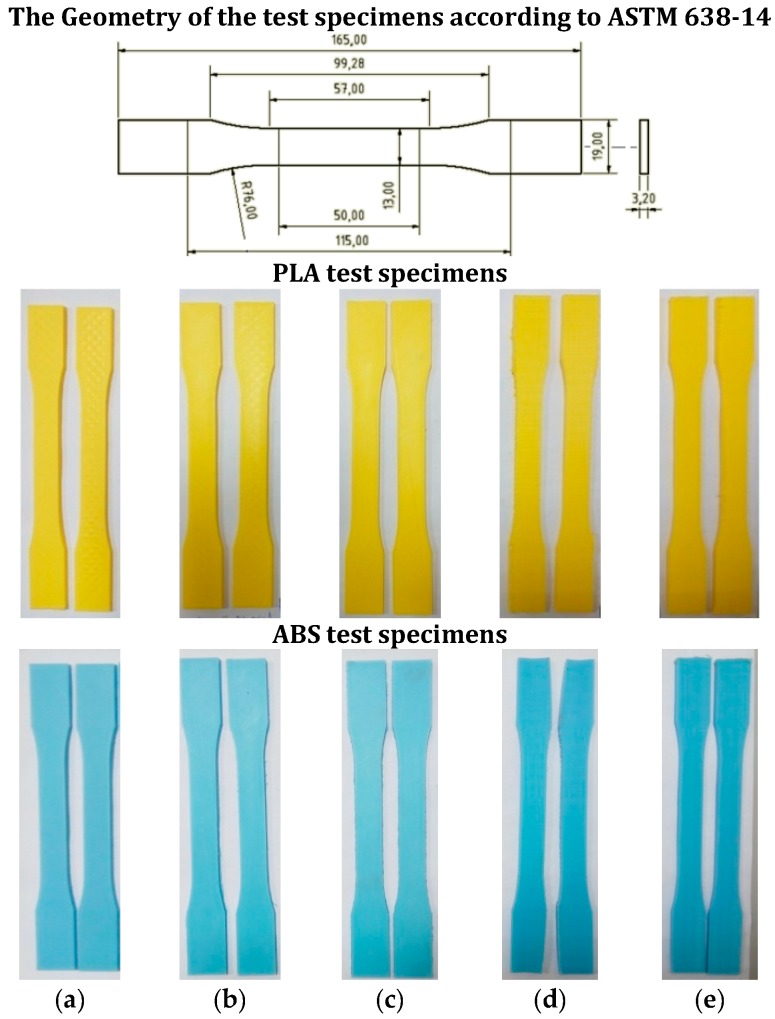
The test specimens manufactured using PLA and ABS. (**a**) Case 1; (**b**) Case 2; (**c**) Case 3; (**d**) Case 4; (**e**) Case 5.

The test procedure that is going to be followed is explained in a previous work [43] and uses the type I general usage test specimen as per ASTM 638-14 [23] (Figure 5, above), as explained in detail in that study. It also includes the principal difficulties encountered during the testing of plastic material specimens obtained using FDM and the fact that the test specimen breaks prematurely due to the concentration of stresses, which have also been reported by other authors, such as Wendt et al. [44]. The ASTM 638-14 standard, indicated by Dizon et al. [45], is the most used for this type of mechanical characterisation. The specimen is fixed by the clamps, aligning its longitudinal axis with the axis of the test machine. The wedges should be tightened enough to avoid the specimen to slide without causing it to collapse. The HOWIN software is used to control the test, to set the input parameters and to gather the values obtained. The speed of the test should be 5 mm/min according to the ASTM D638-14 standard (the minimum value of those established in the standard that causes the break between 0.5–5 min).

According to the ASTM 638-14 standard, the nominal tensile stresses must be used, that is, the tensile load carried by the test specimen at any given moment per unit area of the minimum original cross section. Since this standard is used for determining the tensile properties of solid plastics obtained for traditional processes, the minimum original cross section is computed by multiplying the thickness of the sample and the width of the narrow section.

Two identical test specimens of each type are going to be manufactured and, in the event of the results varying significantly, a third test shall be carried out. The elongations will refer to the variation of the distance between grips and not to the variation of the benchmark length measured using an extensometer, indicated in standard ISO 527-1:2012 [46], which recommends the use of the nominal distortion when extensometers are not used. Finally, a velocity of 5 mm/min is going to be set in accordance with that established in the ASTM standard, so that the rupture occurs between 0.5 and 5 mins. Figure 5 shows examples of the PLA and ABS test specimens used in the tests arranged by case.

The variables to be studied are tensile yield stress (*R*_p_), mechanical tensile strength (*R*_m_), nominal strain at break (ε_t_), and the modulus of elasticity (*E*_t_).

## 3. Results

### 3.1. Mechanical Behaviour of the PLA Parts

**Figure 6:**
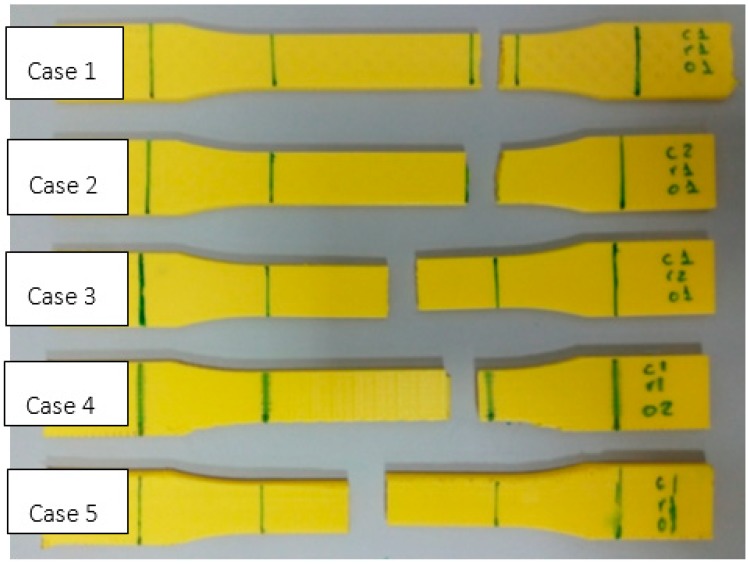
The PLA test specimens tested (one of each type).

Figure 6 shows the PLA test specimens after the test, with a specimen of each type being included. Although all of them fracture in the narrow area, it can be seen that some do so outside the limits of the benchmark length. This is one of the difficulties experienced during the laboratory tests described in a previous study [35].

In the first two cases, a rupture with an irregular breakage line can be observed. This is due to the layers being oriented in the direction of the stress and the raster angle of the infill being 45°. In the third case (50% infill), the test specimen behaves almost as though it were solid, as can be observed in its transversal cross-section. In the fourth case (orientation 2), the line of fracture occurs along a line perpendicular to the stress and located at a point where the infill joins with the walls. In the last case (orientation 3), the fracture occurs as a result of the layers separating because they were built up perpendicular to the stress due to the construction orientation.

**Figure 7:**
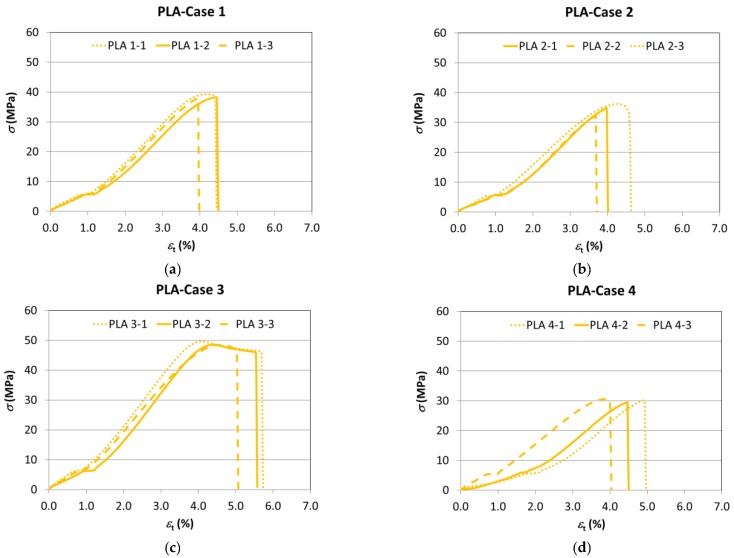

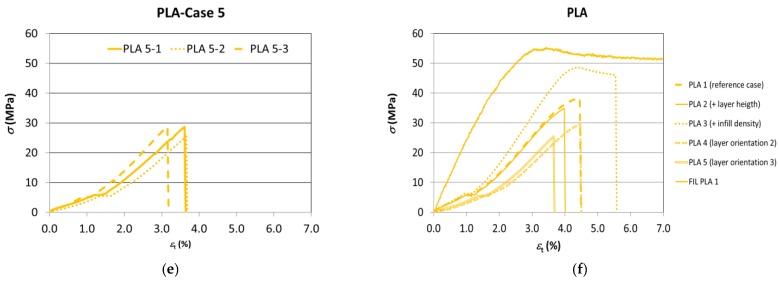
The stress–strain curves (PLA). (**a**) Case 1; (**b**) Case 2; (**c**) Case 3; (**d**) Case 4; (**e**) Case 5; (**f**) the comparison of the results with the intermediate values of each series of test specimens.

The graphs in Figure 7 show the test results for the different parameters. To help analyse these data more clearly, a graph is provided showing the test data for each type of test specimen grouped together along with the properties of the filament (Figure 7f). In each case, the results showing the intermediate values of each series have been chosen.

The corresponding mechanical properties of the PLA test specimens have been calculated based on the information gathered during the tests. The data corresponding to the yield stress, tensile strength, nominal strain at break, and the modulus of elasticity are shown in Table 6.

Firstly, by comparing the results with respect to the benchmark case (Case 1), it is found that, indeed, the variation of the manufacturing parameters causes a variation in the tensile behaviour of the PLA test specimen, with its general performance being on the fragile side compared with that of the filament, which presents a high degree of ductility.

The slope of the elastic area (and, consequently, Young’s modulus) remains practically unmodified by the change in layer height (Case 2), while it clearly diminishes when the manufacturing orientation is modified (Cases 4 and 5), and rises on the infill percentage being increased (Case 3).

The nominal strain at break (and, therefore, the ductility) clearly increases in those cases with a higher infill percentage (Case 3) and also with orientation 2 (Case 4), while it is reduced with manufacturing orientation 3 (Case 5) and remains more or less invariable with the increase of layer height (Case 2).

In all the cases, Young’s modulus and the nominal strain at break are lower than in the filament.

The mechanical strength rises with the increase of infill percentage (Case 3) with respect to the initial conditions (Case 1). Meanwhile, a reduction thereof is observed in the cases of increased layer height (Case 2) and variation of the construction orientation (Cases 4 and 5), with this reduction being especially noteworthy in the case of orientation 3 (Case 5), as was to be expected.

Finally, the yield stress presents a performance similar to the tensile strength, bearing in mind that they practically coincide in many cases given the generally fragile performance of the PLA test specimens.

Considering mechanical strength to be the parameter of greatest interest due to its greater repeatability across laboratories and to its importance as a mechanical property from the design and functionality point of view for certain applications, Table 7 shows the percentage variation of the average mechanical strength in terms of the parameters used and the variation with respect to the material of the filament (data provided by the manufacturer).

**Figure 8:**
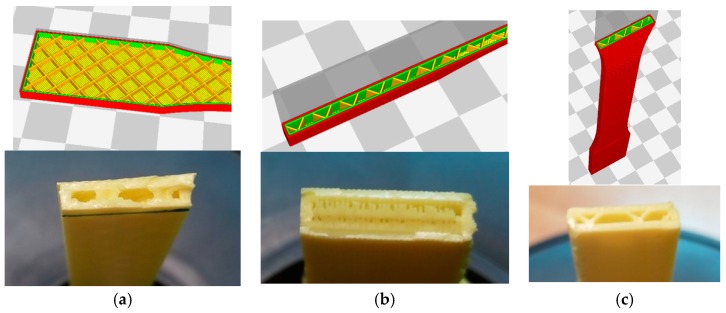
The detail of the infill and fracture surface in terms of orientation. (**a**) Orientation 1; (**b**) Orientation 2; (**c**) Orientation 3.

These data reveal that increasing the infill up to 50% (Case 3) greatly improves the mechanical strength (27%) as this makes the part more solid and reduces the number of cavities (its weight increases by 16%). The increase of layer height (Case 2) causes the maximum tensile strength to fall by 11%. Although this effect is less pronounced than that of varying the infill and the manufacturing orientation, it can be seen that finer layers lead to better results as far as both the finish and mechanical properties are concerned. With respect to the manufacturing orientations, orientation 2 (Case 4), with the layers in a direction parallel to the stress, but perpendicular to those of orientation 1, reduces the mechanical tensile strength by 22% (Figure 8a). On observing the fractured part (Figure 8b), it can be seen that this behaviour might be due to the different orientation of the infill with respect to the direction of the stress, which is what causes the part to rupture at the point where the infill joins the wall.

In the case of orientation 3 (Case 5), the overlap of layers perpendicular to the direction of the tensile stress causes the tensile strength to fall by 28%. This reduction of strength caused by varying the manufacturing orientation is due to the anisotropy of the pieces manufactured in the layers. In Case 5, this orientation causes the stress to revert to the interface between the layers, namely the weakest area of the pieces produced using additive manufacturing (Figure 8c).

In the table, it can also be seen that with the best of the combinations of parameters (Case 3) it is possible to achieve a strength that is only 4% less than that specified for the filament by the manufacturer.

### 3.2. Mechanical Behaviour of the ABS Pieces

**Figure 9:**
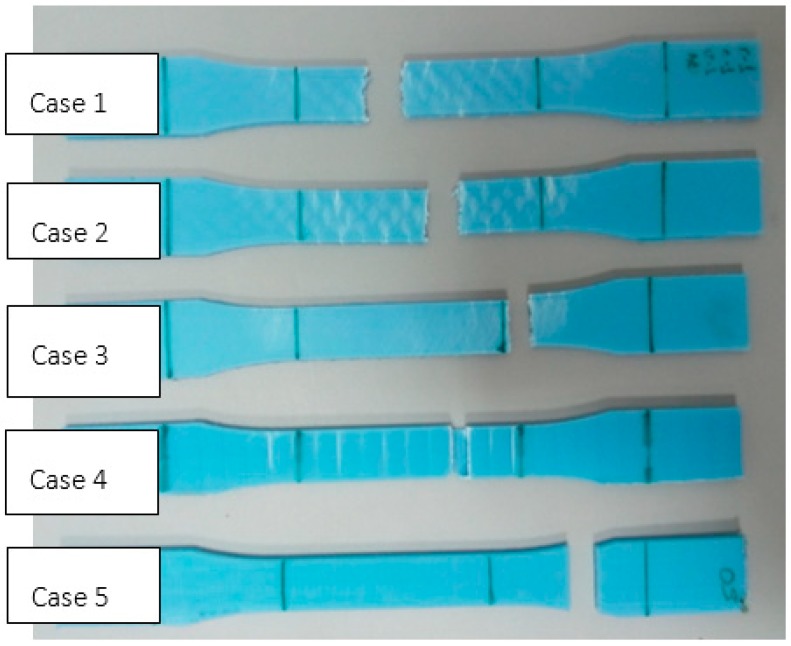
The ABS test specimens tested (one of each type).

Figure 9 shows the ABS test specimens after the test, with a specimen of each type being included. It can be observed that all the test specimens rupture where they are narrowest (some of them outside the benchmark length) except the one manufactured with orientation 3 (layers perpendicular to the stress), which ruptured with an extremely low stress due to a separation of layers in the area of the agreed radius.

**Figure 10:**
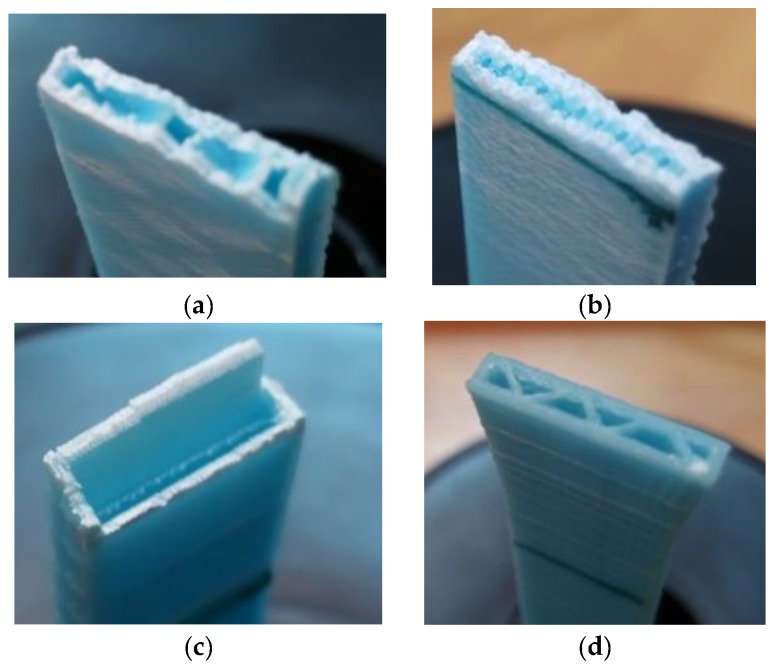
The fracture cross-section. (**a**) Case 1; (**b**) Case 3; (**c**) Case 4; (**d**) Case 5.

In Cases 1 and 2, a rupture with a jagged line can be observed. This is due to the layers being oriented in the direction of the stress and the raster angle of the infill being 45° (Figure 10a). In Case 3, given that it has 50% of infill, the test specimen is almost solid, which is why the distortion is more uniform (Figure 10b). In Case 4, the fracture occurs along lines perpendicular due to the stress and located at the points where the infill joins the walls (Figure 10c). In Case 5, the fracture occurs at low distortions as a result of the separation of layers because, due to the construction orientation these build up perpendicular to the stress, which causes the test specimen to break prematurely as the interface between the layers in this material is extremely weak (Figure 10d).

**Figure 11:**
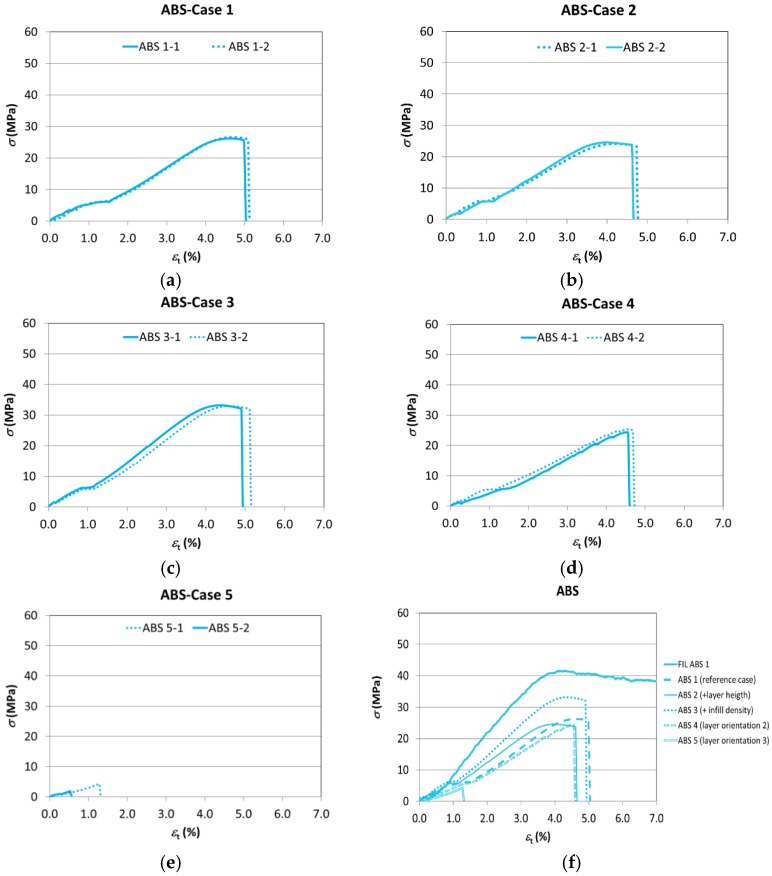
The stress–strain curves (ABS). (**a**) Case 1; (**b**) Case 2; (**c**) Case 3; (**d**) Case 4; (**e**) Case 5; (**f**) The comparison of the results with the intermediate values of each series of test specimens.

The graphs of Figure 11 show the results of the tests with the different parameters for the test specimens manufactured using ABS, where a lower variability in the results than in the case of the PLA test specimens can be observed.

The data corresponding to the yield stress, tensile strength, nominal strain at break, and modulus of elasticity are shown in Table 8.

It can be seen how the change of manufacturing parameters also causes a variation in the tensile performance of the ABS test specimens.

The slope of the elastic area (stiffness) remains almost the same when orientation 2 is used (Case 4), while said incline (and, therefore, Young’s modulus) increases significantly with an infill of 50% (Case 3), and less noticeably with an increase in layer height (Case 2). As regards orientation 3 (Case 5), the test specimen breaks so quickly that it is hardly possible to draw conclusions as to its tensile performance except for the fact that its deformation capacity is practically nil.

The nominal strain at break is slightly affected by layer height (Case 2), nor by the change to orientation 2 (Case 4) or by the increased percentage of infill (Case 3). However, the strain at the break diminishes significantly with respect to Case 1 when orientation 3 is used (Case 5).

In all the cases, Young’s modulus and strain at break are lower than in the filament, as would occur in the case of PLA.

On the other hand, with the exception of the cases of vertical orientation (Case 5) and the increase of the infill (Case 3), the differences with regards to mechanical strength are not too pronounced in all the other cases. Both the use of thicker layers (Case 2) and the change of the layer orientation to keep them parallel to the stress (Case 4) reduce the maximum strength, but only slightly. Increasing the infill to 50% (Case 3), with the resulting 18.5% rise in weight, causes the tensile strength to increase by 25.19%, while orienting the layers perpendicularly to the stress (Case 5) causes the premature rupture of the test specimen with a strength reduction of 88.07% (Table 9). Another interesting fact shown by the table is that with the different combinations of parameters, the best results that can be obtained are 22% worse than those of the filament.

The change of infill results in a more solid test specimen and a reduction of cavities, once again result in an increase of mechanical strength. As regards layer orientation, if this is perpendicular to the stress, it causes the strain to revert to the interface between layers, namely the weakest area in the pieces obtained using additive manufacturing and especially in the case of ABS. The weakness of this interface can be seen in the graph, which shows that the piece breaks under a far lower strain than the pieces manufactured using other orientations due to layer separation.

### 3.3. Comparative Analysis

**Figure 12:**
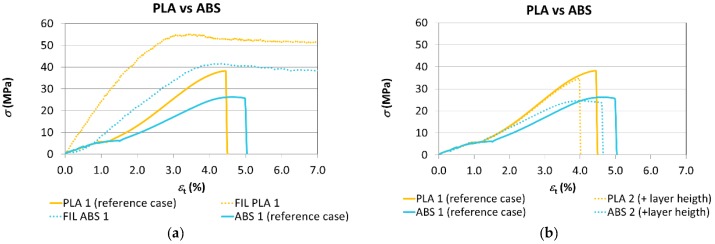

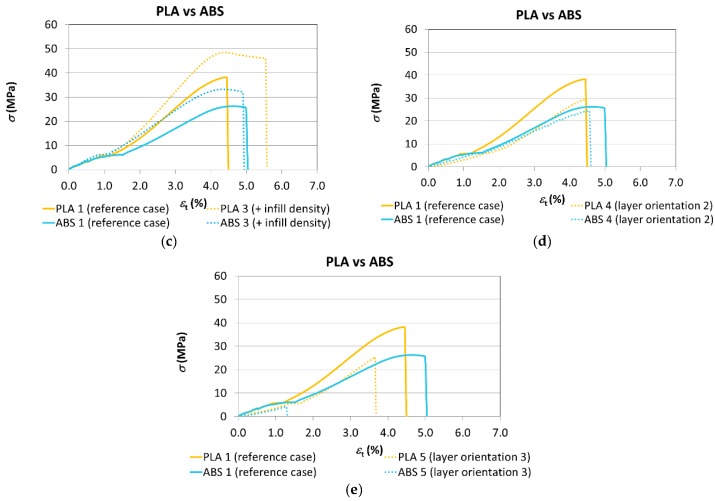
The comparison of the stress–strain curves for both materials. (**a**) Filaments vs. Reference case, (**b**) Reference case vs. + layer height, (**c**) Reference case vs. + infill density, (**d**) Reference case vs. layer orientation 2, (**e**) Reference case vs. layer orientation 3.

In the stress-strain graph of Figure 12, the tensile performance of both materials is shown together to facilitate the comparative analysis.

**Figure 13:**
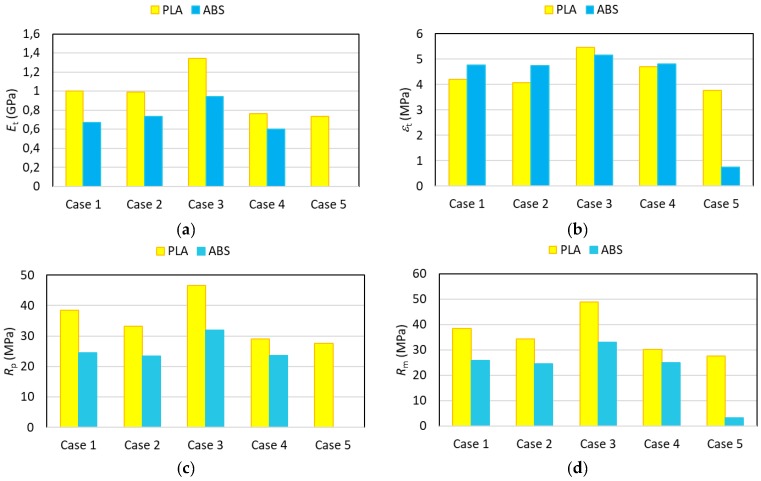
The comparison of mechanical properties for PLA and ABS. (**a**) Modulus of elasticity; (**b**) Nominal strain at break; (**c**) Yield stress; (**d**) Tensile strength.

The variability of the results can be compared for both materials through Table 6 and Table 8 through the standard deviation. The variability of the modulus of elasticity is very similar for PLA and ABS, although, in general, it can be stated that results with ABS showed a lower variability than in the case of PLA. To help quantify the effect of the manufacturing parameters, Figure 13 shows the data corresponding to the modulus of elasticity, nominal strain at break, yield stress, and tensile strength obtained following the tests for the 5 cases analysed.

Both figures show that the test specimens manufactured using PLA generally present a more rigid performance associated with the higher values of Young’s modulus found, in line with the values obtained for the filament. The influence of the manufacturing parameters presents a similar trend for both materials.

As far as the strain at break values obtained are concerned, it can be said that there is no clear trend given that, for example, in Cases 1 and 2, a greater ductility is obtained for ABS, while in Cases 3 and 5 the PLA’s ductility is clearly higher, with very similar values being obtained for both materials in Case 4.

On the other hand, in light of the values obtained, the pieces manufactured using PLA have a greater tensile strength than their ABS counterparts, just as would occur with the filament. Furthermore, the change of parameters has less of an influence on mechanical strength in the case of ABS, with the exception being Case 5, where the reduction of mechanical strength is more significant. Figure 13d shows that PLA undergoes more significant reductions of maximum strength than ABS when layer height is increased (Case 2) and transversal orientation is used (Case 4). Under all circumstances, the infill percentage (Case 3) is the factor with the greatest influence on the results. The performance of the tensile yield stress is identical to that of the maximum strength.

Finally, the differences with respect to the starting material and the decrease of mechanical strength in the case of orienting the layers perpendicular to the stress (Case 5) are less in the PLA, which would appear to indicate a significantly stronger bond of the layers in this material (Table 10).

Based on this data, it can be observed that increasing the infill up to 50% (Case 3) causes a marked increase in mechanical strength, with this being of a similar magnitude in both materials (27% PLA + 16% weight and 25% ABS + 18% weight). This is because the piece is more solid and has fewer cavities.

As has already been explained, the layer height effect (Case 2) is more significant with PLA as it causes an 11% reduction in tensile strength, whereas in the case of ABS this is only 8%. It can thus be seen that finer layers lead to better results not only where the finish is concerned, but also in mechanical properties, although the effect is more noticeable in the PLA than in the ABS.

As far as the manufacturing orientations are concerned, orienting the layers in the direction parallel to the stress, but perpendicular to Case 1 (Case 4), greatly reduces tensile strength in PLA, (−22%) while its effect is not remarkable in ABS (−6%).

In the case of vertical orientation (Case 5), the overlapping of layers perpendicular to the direction of the tensile force reduces tensile strength by 28% in PLA, a much lower figure than in the case of ABS, which fractures prematurely (with an 88% reduction of maximum strength) due to its weak bond between layers.

The variations with respect to the filament lead to the conclusion that the material which undergoes a lower reduction of its mechanical properties during the additive manufacturing process is PLA (a mere 25% as against the 38% of ABS under benchmark conditions), which would appear to indicate the bond it achieves between layers is better than that of ABS, thus, making it an ideal thermoplastic for use in FFF/FDM technologies.

Finally, in Table 10 it can be observed that with the best combination of parameters it is possible to achieve a strength of only 4% less than that specified by the manufacturer for the PLA material, namely, figures that are far better than those returned by ABS (−22%). This indicates that the bond between layers in this material is far better than in ABS, at least with the manufacturing parameters used in this study, and that with an optimum combination of parameters (a 100% infill, the alignment of layers in the direction of the stress, a layer height of 0.1 mm, and optimum raster and pattern, temperature and velocity parameters), it could be possible to achieve strength results very similar to those of the starting filament or injection or compression moulded pieces.

## 4. Conclusions and Future Work

This work has studied the influence of the principal manufacturing parameters of an FDM process on the mechanical properties of the two thermoplastic materials most widely used in this type of additive technique: ABS and PLA. These parameters are infill percentage, layer height and manufacturing orientation. The results with ABS show a lower variability than in the case of PLA.

Generally speaking, the pieces manufactured with PLA behave more rigidly than in the case of ABS, just as with the starting material, with the influence of the manufacturing parameters being very similar for both materials. However, there is no clear trend regarding the influence of the manufacturing parameters on the strain at fracture values obtained, unlike the starting material, where the ABS showed a higher degree of ductility than the PLA.

Regarding ABS, the mechanical strength results barely vary with respect to layer height (the maximum strength only falls by 7.57% when the layer height is increased from 0.1 to 0.2 mm) and when using another orientation in which the layers remain parallel to the stress (a reduction of tensile strength by an average of 5.87%). However, increasing the infill up to 50% (with an 18.5% weight increase) causes the tensile strength to rise by 25.19% given that the test specimen is more solid and the cavities are smaller. On the other hand, the vertical orientation of the layers perpendicular to the stress causes the premature rupture of the test specimen with a strength reduction of 88.07%. This is due to the strain reverting to the interface between layers, namely, the weakest area in the pieces obtained using additive manufacturing, especially in the case of ABS.

Another interesting point to emerge from this study is that, with the different combinations of parameters chosen, the best results that can be obtained are 22% worse than those of the starting material. This, combined with the lack of strength of the test specimens whose layers are oriented perpendicularly to the stress, confirms that the ABS pieces manufactured using this technology and these parameters show an extremely weak bond compared with the strength of the material.

In the case of PLA, the increased layer height causes the tensile strength to diminish by 11%, which is notably greater than in the case of ABS, which is why it can be concluded that finer layers lead to better results not only where finish is concerned, but also in mechanical properties, although the effect is more noticeable in the PLA than in the ABS. On the other hand, it can be seen that increasing the infill up to 50% (with a 16% rise in weight), greatly improves mechanical strength (27%), which is very similar to that of ABS (25.19%).

With respect to the manufacturing orientations of the layers in a direction parallel to the stress, but perpendicular to those of the initial orientation, the tensile strength is reduced by 22%, far and above that of ABS (5.87%). This behaviour can be attributed to the different orientation of the infill with respect to the direction of the stress, which is what causes the part to fracture at the point where the infill joins the wall.

The orientation with the overlapping of layers perpendicular to the tensile force the reduces maximum tensile strength by 28%, a much lower figure than in the case of ABS, which fractured prematurely (with an 88.07% reduction of the maximum strength) due to its weak bond between layers.

Regarding PLA it can be concluded that, with the best of the combinations of parameters a strength of only 4% less than that specified by the manufacturer for the material can be achieved, which is far better than that returned by the ABS (−22%). This indicates that the bond between layers in this material turns out to be extremely strong and is, therefore, highly suitable for use in additive technologies given that with an optimum combination of parameters it is possible to achieve strength results very similar to those provided by injection or compression moulded pieces.

In general, the infill percentage is the factor of greatest influence in the results. The performance of the yield stress is identical to that of the maximum strength.

The methodology proposed is a reference of interest in studies involving the determination of mechanical properties of polymer materials manufactured using these technologies. Specifically speaking, these results can be extremely useful for the selection of suitable materials and parameters in FDM design and manufacturing processes.

The inclusion of other factors of interest such as the direction of the raster and manufacturing speed, as well as the influence of the interaction of these factors on the results, is proposed as a line of work for the future. Additionally, the influence of other advanced parameters that are closely related to more specific thermophysical and/or chemical phenomena (the local temperature of the filament during deposition, the molecular diffusion at the polymer interface, and the deposition pressure/force, among others) will be also addressed in future studies; this kind of analysis will require specialised equipment in most of the cases due to its complex nature.

## Figures and Tables

**Table 1:** The mechanical tensile properties of the filaments employed according to the manufacturer.

Material (Manufacturer)	Colour	Tensile Strength (MPa)	Nominal Strain at Break (%)	Modulus of Elasticity (GPa)	Density (g/cm^3^)
PLA (BQ)	Yellow	51	6	3.5	1.25
ABS (PrintedDreams)	Blue	41–45	20	2.1	1.05

**Table 2:** The technical characteristics of the FDM equipment.

Characteristic	Value
Maximum printing volume (mm^3^)	215 (*x*) × 210 (*y*) × 180 (*z*)
Firmware	Marlin
Nozzle diameter (mm)	0.4
Resolution (mm)	0.1
Maximum printing velocity (mm/s)	80
Maximum displacement velocity (mm/s)	120

**Table 3:** The comparison of mechanical properties (laboratory vs. manufacturer).

Material	Average Tensile Strength (MPa)	Manufacturer Tensile Strength (MPa)	Difference (%)	Average Nominal Strain at Break (%)	Manufacturer Nominal Strain at Break (%)	Difference (%)	Average Modulus of Elasticity (GPa)	Manufacturer Modulus of Elasticity (MPa)	Difference (%)
PLA	55.80	51.0	+9.4	12.75	6	+112.5	1.94	3.5	−44.6
ABS	42.35	42.5	−0.4	19.58	20	−2.1	1.49	2.1	−29.0

**Table 4:** The definition of case studies for a layer width of 0.4 mm, a printing velocity of 60 mm/min (30 mm/min on wall and bases), a line-type wall pattern, and infill patterns of 45° and 135°.

Case	Layer Height (mm)	Infill (%)	Manufacturing Orientation
Case 1 (Reference)	0.1	20	Orientation 1
Case 2	0.2	20	
Case 3	0.1	50	
Case 4	0.1	20	Orientation 2
Case 5	0.1	20	Orientation 3

**Table 5:** The remaining manufacturing parameters specified for each material.

Material	Fusor Temp. (°C)	Base Temp. (°C)	Wall Vel./Infill (mm/s)	Layer Width (mm)	Adhesion Plat.	Vent. Layer	Dist./Vel. Retraction (mm/mm/s)	Wall/Infill Pattern and Angle (°)
ABS	235	80	60/30	0.4	YES (8 mm)	100%	6/25	Lines/grid (45, 135)
PLA	200	50			NO			

**Table 6:** The mechanical properties of PLA test specimens and the variation with respect to the benchmark case (Case 1).

Case	E_t_ (GPa) Mean (St. Dev.)		ε_t_ (%) Mean (St. Dev.)		R_p_ (MPa) Mean (St. Dev.)		R_m_ (MPa) Mean (St. Dev.)	
Case 1	1.000 (0.02)		4.20 (0.30)		38.43 (0.68)		38.47 (0.74)	
Case 2	0.987 (0.03)	≅	4.07 (0.50)	≅	33.13 (1.19)	−	34.37 (2.08)	−
Case 3	1.343 (0.08)	++	5.47 (0.32)	++	46.53 (2.27)	++	48.87 (0.64)	++
Case 4	0.760 (0.11)	−	4.70 (0.72)	+	29.03 (0.70)	−−	30.10 (0.56)	−−
Case 5	0.735 (0.02)	−	3.77 (0.21)	−−	27.63 (1.85)	−−	27.63 (1.85)	−−

≅ (similar to the benchmark case), + (higher than the benchmark case), ++ (much higher than the benchmark case), − (lower than the benchmark case), −− (much lower than the benchmark case).

**Table 7:** The comparison of the mechanical properties of PLA test specimens with those of the filament.

Case	Mean *R*_m_ (MPa)	Difference to Case 1 (Benchmark)	Difference to Filament
Case 1	38.47	−	−25%
Case 2	34.37	−11%	−33%
Case 3	48.87	+27%	−4%
Case 4	30.10	−22%	−41%
Case 5	27.63	−28%	−46%

**Table 8:** The mechanical properties of the ABS test specimens and variation with respect to the benchmark case (Case 1).

Case	E_t_ (GPa) Mean (St. Dev.)		ε_t_ (%) Mean (St. Dev.)		R_p_ (MPa) Mean (St. Dev.)		R_m_ (MPa) Mean (St. Dev.)	
Case 1	0.650 (0.01)		5.10 (0.00)		25.60 (0.00)		26.40 (0.28)	
Case 2	0.732 (0.02)	+	4.75 (0.07)	−	23.45 (0.35)	−	24.40 (0.28)	−
Case 3	0.943 (0.11)	++	5.15 (0.21)	≅	31.95 (0.07)	++	33.05 (0.21)	++
Case 4	0.600 (0.03)	≅	4.80 (0.00)	≅	23.60 (1.27)	−	24.85 (0.64)	−
Case 5	−		0.75 (0.35)	−−	3.15 (1.77)	−−	3.15 (1.77)	−−

≅ (similar to the benchmark case), + (higher than the benchmark case), ++ (much higher than the benchmark case), − (lower than the benchmark case), −− (much lower than the benchmark case).

**Table 9:** The comparison of the mechanical properties of ABS test specimens with those of the filament.

Case (ABS)	Mean *R*_m_ (MPa)	Difference to Case 1 (Benchmark)	Difference to Filament
Case 1	26.40	−	−38%
Case 2	24.40	−7.57%	−43%
Case 3	33.05	+25.19%	−22%
Case 4	24.85	−5.87%	−42%
Case 5	3.15	−88.07%	−93%

**Table 10:** The comparison of mechanical strength with the benchmark case and the filament.

Material	Case 1	Case 2	Case 3	Case 4	Case 5
PLA	38.47	34.37	48.87	30.10	27.63
Difference to Case 1	−	−11%	+27%	−22%	−28%
Difference to filament	−25%	−33%	−4%	−41%	−46%
ABS	26.40	24.40	33.05	24.85	3.15
Difference to Case 1	−	−8%	+25%	−6%	−88%
Difference to filament	−38%	−43%	−22%	−42%	−93%
